# Aberration correction considering curved sample surface shape for non-contact two-photon excitation microscopy with spatial light modulator

**DOI:** 10.1038/s41598-018-27693-7

**Published:** 2018-06-18

**Authors:** Naoya Matsumoto, Alu Konno, Takashi Inoue, Shigetoshi Okazaki

**Affiliations:** 1 Central Research Laboratory, Hamamatsu Photonics K.K, Hamamatsu, Japan; 2 Department of Medical Spectroscopy, Institute for Medical Photonics Research, Preeminent Medical Photonics Education & Research Center, Hamamatsu University School of Medicine, Hamamatsu, Japan

## Abstract

In this paper, excitation light wavefront modulation is performed considering the curved sample surface shape to demonstrate high-quality deep observation using two-photon excitation microscopy (TPM) with a dry objective lens. A large spherical aberration typically occurs when the refractive index (RI) interface between air and the sample is a plane perpendicular to the optical axis. Moreover, the curved sample surface shape and the RI mismatch cause various aberrations, including spherical ones. Consequently, the fluorescence intensity and resolution of the obtained image are degraded in the deep regions. To improve them, we designed a pre-distortion wavefront for correcting the aberration caused by the curved sample surface shape by using a novel, simple optical path length difference calculation method. The excitation light wavefront is modulated to the pre-distortion wavefront by a spatial light modulator incorporated in the TPM system before passing through the interface, where the RI mismatch occurs. Thus, the excitation light is condensed without aberrations. Blood vessels were thereby observed up to an optical depth of 2,000 μm in a cleared mouse brain by using a dry objective lens.

## Introduction

The observation of a given biological sample with cellular-level resolution is expected to be performed for the investigation of biological functions. Recently, confocal microscopy^1,2^, light-sheet microscopy^3,4^, and two-photon excitation microscopy (TPM)^5–7^ were proposed. TPM provides a high-resolution image in the deep region of the biological sample owing to lower amounts of scattering and out-of-focus fluorescence. As TPM observation examples, morphological observations of nerve fibres from the surface of a mouse to its hippocampus^8^, and the contribution of the glial cells to the neural circuit^9^ have been performed *in vivo*. Other body parts, including the kidney^10^, spinal cord^11,12^, skin^13^, and bone marrow^14^, have also been observed.

Various methods for reducing the effects of scattering, absorption, and aberration were proposed for observing deeper regions of biological samples. Kobat *et al*. reduced the influence of scattering by setting the excitation light wavelength from 775 nm to 1280 nm, thereby realising the observation of vasculature at a depth of 1 mm^15^ *in vivo*. By introducing a clearing technique^16–20^ in an *in vitro* experiment, scattering, absorption, and aberration were reduced, and observation at a depth of 6 mm or more was realised with a special objective lens for cleared samples^18^.

The main contributing factors for the occurrence of aberrations are the internal structure and surface shape of the biological sample, as well as the refractive index (RI) mismatch between the immersion medium and the biological sample. In particular, when a dry objective lens is used, the curved surface shape and large RI mismatch between air and the sample strongly generate lower-order aberrations of tilt, defocus, astigmatism, coma, and spherical aberrations. Generally, to reduce the influence of the RI mismatch, an immersion-fluid objective lens is used, where the immersion fluid fills the space between the objective lens and the sample. In an observation using an upright microscope, a piece of equipment, such as a glass-bottom dish, is placed on the sample for infilling with the immersion fluid^8,11^. An additional effective approach involves pressing a glass-bottom dish (or a glass cover) on the sample so that the interface between air and the sample becomes perpendicular to the optical axis and spherical aberration becomes dominant. It is thereby possible to correct the aberration using an objective lens correction collar.

Nevertheless, the observation method using the immersion-fluid objective lens has disadvantages. Preparations for attaching the dish must be completed before the observation, and the dish-pressing may cause stress in a living sample. Additionally, when a highly viscous immersion fluid is used for tissue clearing, large aberrations may be generated owing to the non-uniformity of the RI distribution (Schlieren phenomena) and bubbles introduced in the fluid. Consequently, a poor-quality image may be obtained, even on the surface of the sample. Moreover, the optical elements may become contaminated if oil is employed as the immersion fluid. Of course, even in the case of observation with an immersion-fluid objective lens, aberrations occur when observing the deep regions if any RI difference exists between the immersion fluid and the sample.

Another approach is adaptive optics (AO), which reduces aberrations due to the internal structure and surface shape of the sample by using a spatial light modulator (SLM). There are two cases in the calibration for correction with AO: with and without a wavefront sensor. In the AO with a wavefront sensor^21–23^, the fluorescence wavefront of a structure having a known shape—often called a “guide star”—is measured by the sensor, and the excitation light wavefront is modulated by the SLM so that the guide-star fluorescence intensity is maximised. In the area around the guide star, the excitation light condenses without aberrations, thereby improving the fluorescence intensity. However, if there is no suitable endogenous structure that can be used as a guide star, one may be implanted at the observation depth in the sample by a surgical operation^21^. On the contrary, in AO without a wavefront sensor^24–28^, multiple scans are performed to adjust the coefficient of each Zernike mode in the calibration to maximise the fluorescence intensity of the object of interest in a measurement image. As multiple scans are carried out for the calibration at each depth, reduction in the number of scans leads to significant decrease in measurement time.

The sample preparation for an observation using a dry objective lens, i.e., a non-contact observation, is much simpler than that using an immersion-fluid objective lens. In addition, observation that does not involve pressing with a glass-bottom dish or implanting guide stars can reduce invasion into the sample. However, the observation-limit depth of a dry objective lens is shallower than that of an immersion-fluid objective lens because of a large RI mismatch between air and the sample.

In this paper, we incorporate a liquid crystal on a silicon-type SLM^29^ into a TPM system for aberration correction considering the curved surface shape, and we realise non-contact and low-invasive observation with a dry objective lens. As the basis of our strategy, aberrations caused by the RI mismatch and the sample surface shape are calculated by a novel, simple optical path length difference (OPD) calculation method to design the pre-distortion pattern. Moreover, the excitation light wavefront is modulated by the SLM so that the excitation light is converged at the desired depth without aberrations. In the observation of the blood vessels of a cleared mouse brain, we observed the vessels up to an optical depth of 2,000 μm. The depth limit of this observation was determined by the working distance of the objective lens. The proposed method can simplify the sample preparation step and, thereby, the observation procedure.

## Results

### Aberration Correction Method Considering Sample Surface Shape Using OPD Calculation Method

In a two-photon excitation microscope, since the fluorescence intensity and the resolution of the obtained image are affected by the concentration distribution of the excitation light, we only need to correct the excitation light wavefront.

**Figure 1:**
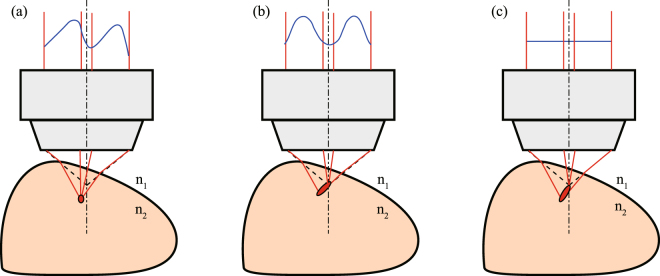
Relationship between the excitation light wavefront and the excitation light concentration in a biological sample. (**a**) Proposed aberration correction method considering the curved sample surface shape (ACMSS): the excitation light wavefront (indicated by the blue line) is modulated by an SLM with consideration of the sample surface shape and is irradiated. (**b**) Conventional method 1: spherical aberration correction method (SACM) when the RI interface between air and the sample is a plane perpendicular to the optical axis. (**c**) Conventional method 2: the excitation light wavefront is not modulated, i.e., a plane wavefront is irradiated. ${n}_{1}$ is the RI of air, and ${n}_{2}$ represents the average RI of the sample.

We attempted to design the pre-distortion wavefront to correct the aberrations caused by the curved shape of the sample. The designed pre-distortion wavefront pattern for correcting the aberration caused by the curved sample surface shape (Fig. 1(a)) is electrically modulated from a plane wavefront (Fig. 1(c)) by the SLM incorporated into a TPM system. In Fig. 1(c), the excitation light having a plane wavefront is refracted at the surface and focused inside the sample with aberrations. On the other hand, in Fig. 1(a), the excitation light is focused at the desired depth because the excitation light wavefront is modulated in advance from the plane wavefront to the pre-distortion wavefront in which the effects of the light refraction and the OPD are considered.

To date, several spherical aberration correction methods (SACMs) using OPD calculation with and without the sample, for the case in which the interface between air and the sample is a plane perpendicular to the optical axis, have been proposed^30–33^. From these studies, when the ideal focal point of a dry objective lens with a numerical aperture ($\overline{NA}$) moves to the inside of the sample (at a distance $d$ from the interface), the pre-distortion wavefront ${\varphi }_{SA}$ when using SACM^33^ is expressed as

1 $${{\varphi }}_{{SA}}({\rho },{d})=-\frac{2\pi {d}}{{\lambda }}((1+{\mu })\sqrt{{{n}}_{2}^{2}-{(\overline{{NA}}{\rho })}^{2}}-\sqrt{{{n}}_{1}^{2}-{(\overline{{NA}}{\rho })}^{2}}),$$

2 $${\rho }({\eta },{\xi })=\frac{p}{M\ast \overline{NA}}\sqrt{{({\eta }+{{\eta }}_{0})}^{2}+{({\xi }+{{\xi }}_{0})}^{2}},$$

where $\eta$ and $\xi$ are the coordinates on the SLM, ${\eta }_{0}$ and ${\xi }_{0}$ are the coordinates of the centre of the light irradiated on the SLM (in this case, ${\eta }_{0}$ and ${\xi }_{0}$ correspond to the centre of the objective lens), and $\lambda$ denotes the excitation light wavelength. In addition, $\mu$ represents the factor for changing the depth of the focal spot, ${n}_{1}$ is the RI of air, ${n}_{2}$ represents the average RI of the sample, $p$ denotes a pixel pitch of the SLM, and $M$ denotes the magnification of the relay lens systems in the TPM system. By using SACM, the pre-distortion pattern for correcting the spherical aberration is designed, and an effect similar to adjusting the correction collar of the objective lens is realised. However, in the observation of a tilted sample or crown-shaped sample such as a mouse brain, not only spherical aberration but also coma and astigmatism occur because of the sample surface shape. Consequently, SACM cannot completely eliminate the aberration, and the excitation light is focused inside the sample with aberrations (Fig. 1(b)).

Generally, when the interface between air and the sample is not a plane perpendicular to the optical axis, the light refraction is calculated using three-dimensional Snell’s law and the normal vector at the intersection point of the sample and the ray forming the excitation light^34^. Therefore, computational complexity is significantly higher compared to the above-mentioned equations.

We derived the aberration correction method considering the curved sample surface shape (ACMSS) in order to realise a simple OPD calculation method. The tilted pre-distortion wavefront was designed using ACMSS so that the tilt of the diffracted excitation light caused by the tilted wavefront is incident along the tilt of the normal vector at the intersection point of the sample and the ray forming the excitation light. For instance, in the observation of the sample, which was simply tilted with respect to the plane perpendicular to the optical axis, the aberrations could be corrected by shifting the position of the pre-distortion wavefront designed by SACM. By moving the centre position of the pre-distortion wavefront according to the tilt of the normal vector with respect to the optical axis, a simple OPD calculation method for the pre-distortion wavefront is realised without three-dimensional Snell’s law. The movement $({\eta }_{1},{\xi }_{1})$ of the pre-distortion wavefront was determined from the specifications of the objective lens, sample tilt, and magnification of the telecentric relay lens system. The pre-distortion wavefront ${\varphi }_{TS}$ for correction of the sample tilted at $\beta$ radians in the $y$-axis direction is expressed as

3 $${{\varphi }}_{{TS}}({\rho }{^{\prime} },{d}{^{\prime} })=-\frac{2\pi d^{\prime} }{\lambda }((1+{\mu })\sqrt{{{n}}_{2}^{2}-{(\overline{{NA}}{\rho }{^{\prime} })}^{2}}-\sqrt{{n}_{1}^{2}-{(\overline{{NA}}{\rho }{^{\prime} })}^{2}}),$$

4 $${\rho }\text{'}({\eta },{\xi },{{\xi }}_{1})=\frac{{p}}{{M}\ast \overline{{NA}}}\sqrt{{({\eta }+{{\eta }}_{0})}^{2}+{({\xi }+{{\xi }}_{0}+{{\xi }}_{1})}^{2}},$$

5 $${d}{\text{'}}_{TS}={d}\,\cos \,{\beta }{,}$$

6 $${{\xi }}_{1}=\frac{\overline{{NA}}\ast f}{{M}\ast p}\ast \frac{{\beta }}{{\sin }^{-1}(\frac{{NA}}{{{n}}_{1}})},$$

where $f$ is the focal length of the objective lens. As the aberration varies according to the focusing depth, the excitation light wavefront is modulated according to the observation depth. Another explanation of our approach is shown in Supplementary Figure 1.

**Figure 2:**
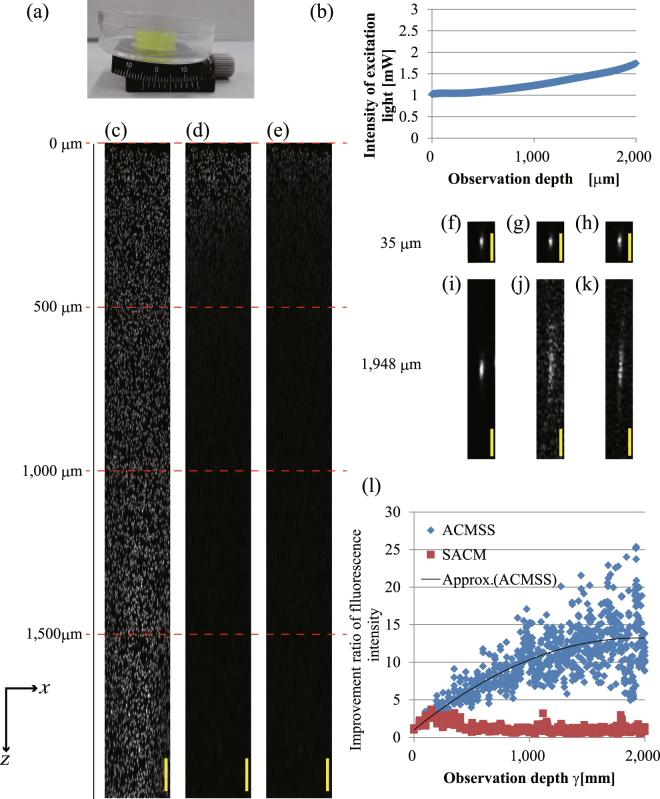
Observation results of fluorescent beads of 3-μm diameter in a transparent epoxy resin tilted at 0.0873 rad (5°). (**a**) Photograph of the transparent epoxy resin tilted by a goniometric rotation stage. (**b**) Excitation light intensity. The excitation light intensity was measured under the objective lens. (**c**–**e**) $xz$-projected images for an optical depth of 0 μm to 2,000 μm obtained from scans performed with wavefront modulation using ACMSS, with wavefront modulation using SACM, and without wavefront modulation, respectively. (**f**–**h**) $xz$ image of the observed bead at a 35-μm optical depth when a TPM scan was performed with wavefront modulation using ACMSS, with wavefront modulation using SACM, and without wavefront modulation, respectively. (**i**–**k**) $xz$ image of the observed bead at a 1,948-μm optical depth. (**l**) Quantitative evaluation of the improvement in the fluorescence intensity from the fluorescent beads. The scale bars indicate 100 μm in (**c**–**e**) and 20 μm in(**f**–**k**).

Figure 2 shows the observation result of 3-μm-diameter fluorescent beads in a transparent epoxy resin (${n}_{2}$ = 1.59) tilted by 0.0873 rad with respect to the $x$-axis. Figure 2(a) shows a photograph of the tilted transparent epoxy resin. We made an oblique plane by using a goniometric rotation stage. To clarify the effect of the aberration correction in the deeper regions, the excitation light intensity was changed with respect to the observation depth (Fig. 2(b)). Figure 2(c) shows the $xz$-projected images obtained using the maximum fluorescence intensity of the beads from an optical depth of 0 μm to 2,000 μm when a TPM scan was performed with wavefront modulation using ACMSS. For comparison, Fig 2(d,e) show the $xz$-projected images when a TPM scan was performed with wavefront modulation using SACM and without wavefront modulation, respectively. The brightness of each image was normalised using the maximum fluorescence intensity of the beads from the scan with wavefront modulation using ACMSS.

Figure 2(c–e) clearly show that the fluorescence intensity of the beads observed near the surface is the highest. The fluorescence intensity of the beads in Fig. 2(d,e) decrease as the observed depth increases. Although the observable depth of the beads in Fig. 2(d) is slightly greater than that in Fig. 2(e), the beads are hardly observed beyond 500 μm. On the other hand, with wavefront modulation using ACMSS, beads are still observed at an optical depth of 2,000 μm in Fig. 2(c). Magnified $xz$-projected images of Fig. 2(c–e) are shown in Supplementary Figure 2.

Figure 2(f–h) show the $xz$ images of the observed bead at an optical depth of 35 μm when a TPM scan is performed with wavefront modulation using ACMSS, with wavefront modulation using SACM, and without wavefront modulation, respectively. Figure 2(i–k) also show the $xz$ images of the observed bead at an optical depth of 1,948 μm. At 1,948 μm, the length of the observed bead with wavefront modulation using the ACMSS is 4.3 times less than that without wavefront modulation. Quantitative evaluation results are shown in Fig. 2(l). At an optical depth of 1,925 μm, we confirmed that the maximum fluorescence intensity with wavefront modulation using the ACMSS is approximately 25 times higher than that without wavefront modulation.

The fluorescence intensity was significantly improved when aberration correction was applied because of the following two reasons. First, in the observation using a dry objective lens, refractive-index mismatch caused a large aberration. Second, the fluorescence is proportional to the square of the excitation light intensity in two-photon excitation fluorescence microscopy.

With the following procedures, it is also possible to correct the aberration caused by an arbitrary sample shape such as a spherical crown shape. First, to elucidate the curved surface of the sample, we performed pre-scanning and measured the auto-fluorescence or fluorescence of the sample. The measured curved surface was approximated to a polynomial. Secondly, we calculated the intersection position of each ray forming the excitation light having the plane wavefront and the sample surface, and we determined the normal vector tilt of the sample at the intersection point. Because the sample had a normal vector with a different tilt at each intersection, the pre-distortion wavefront could be obtained by applying a different tilt in equations (3–6) at each intersection point.

**Figure 3:**
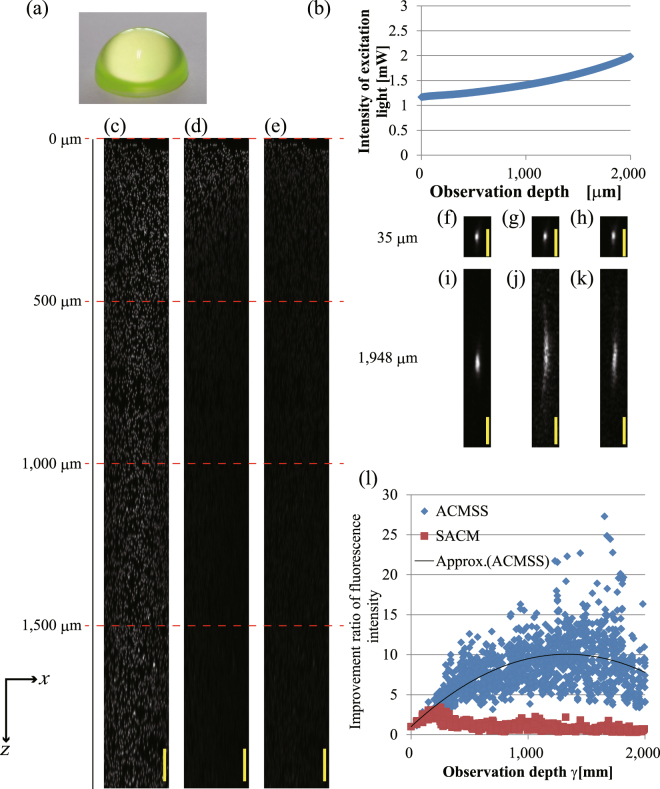
Observation results of fluorescent beads of 3-μm diameter in a spherical-crown-shaped transparent epoxy resin (radius of 6 mm). (**a**) Photograph of the spherical-crown-shaped transparent epoxy resin. (**b**) The excitation light intensity is changed with respect to the observation depth. (**c**–**e**) $xz$-projected images for an optical depth of 0 μm to 2,000 μm from the scans performed with wavefront modulation using the ACMSS, with wavefront modulation using the SACM, and without wavefront modulation, respectively. (**f**)–(**h**) $xz$ image of the observed bead at 35-μm optical depth when a TPM scan is performed with wavefront modulation using ACMSS, with wavefront modulation using SACM, and without wavefront modulation, respectively. (**i**)–(**k**) $xz$ image of the observed bead at 1,948-μm optical depth. (**l**) Quantitative evaluation of the improvement in fluorescence intensity from the fluorescent beads. The scale bars indicate 100 μm in (**c**–**e**) and 20 μm in (**f**–**k**).

Figure 3 shows the observation result of the fluorescent beads in a spherical-crown-shaped transparent epoxy resin. Figure 3(a) shows a photograph of the spherical-crown-shaped transparent epoxy resin (radius of 6 mm). The observation position is centred at a position −500 μm from the top of the spherical crown in the $x$-direction. Figure 3(c–e) show the $xz$-projected images obtained using the maximum fluorescence intensity of the beads from an optical depth of 0 μm to 2,000 μm when a TPM scan is performed with wavefront modulation using the ACMSS, with wavefront modulation using the SACM, and without wavefront modulation, respectively. The magnified $xz$-projected images of Fig. 3(c–e) are shown in Supplementary Figure 3. Figure 3(i–k) also show $xz$ images of the observed bead at an optical depth of 1,948 μm. The observed bead length with wavefront modulation using the ACMSS is 1.8 times less than that without wavefront modulation. The quantitative evaluation results are shown in Fig. 3(l). At an optical depth of 1,816 μm, we confirmed that the maximum fluorescence intensity with wavefront modulation using the ACMSS is approximately 20 times higher than that without wavefront modulation.

**Figure 4:**
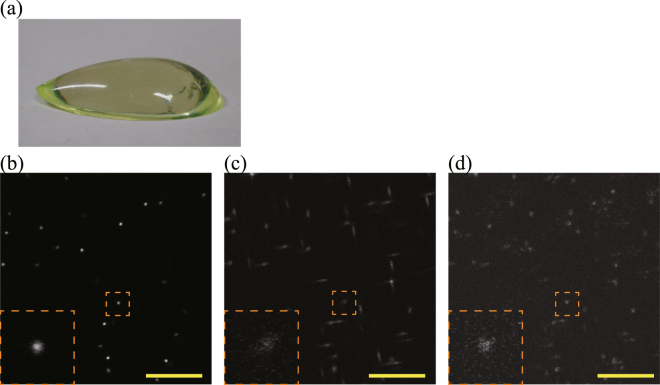
Observation results of 1-um-diameter fluorescent beads in an asymmetric aspheric transparent epoxy resin. (**a**) Photograph of the asymmetric aspheric transparent epoxy resin. (**b**)–(**d**) $xy$ images at an optical depth of 462 μm from the scans performed with wavefront modulation using the ACMSS, with wavefront modulation using the SACM, and without wavefront modulation, respectively. Magnified views of the blood vessels in the orange dashed box are shown in the lower left of (**b**–**d**). The brightness of each image is normalised using the maximum fluorescence intensity of each image. Scale bar indicates 20 μm.

Figure 4 shows the observation results of the 1-μm-diameter fluorescent beads in an asymmetric aspheric transparent epoxy resin. Figure 4(a) shows a photograph of the asymmetric aspheric transparent epoxy resin. Figure 4(b–d) show $xy$ images at an optical depth of 462 μm when a TPM scan is performed with wavefront modulation using the ACMSS, with wavefront modulation using the SACM, and without wavefront modulation, respectively. The condensing positions of sagittal and meridional rays of the excitation light are different owing to the aspheric epoxy resin when TPM scans with wavefront modulation using the SACM and without wavefront modulation were performed. As shown in Fig. 4(c,d), the obtained image was affected by astigmatism and coma. By using ACMSS, it is possible to correct aberrations caused by the asymmetric aspherical surface shape (Fig. 4(d)).

### Observation of Blood Vessels of a Mouse Brain Stained with Fluorescent Dye

**Figure 5:**
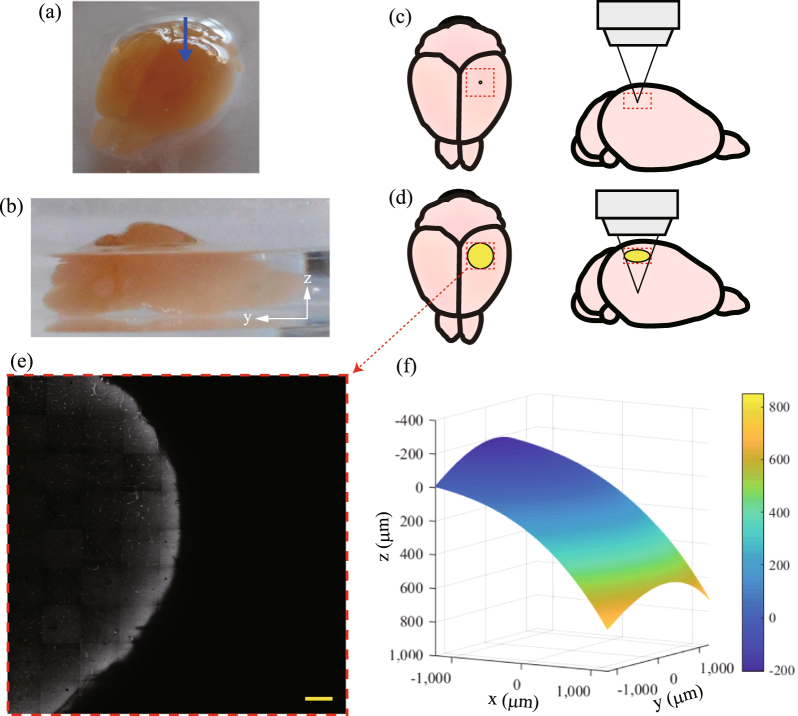
Pre-scanning results of a mouse brain: (**a**,**b**) Photographs of the mouse brain stained by fluorescent dye (DiI). Optical transparency of the brain was enhanced by an optical clearing agent (SeeDB). Measurements were performed near the position indicated by the blue arrow in (**a**). Relationship between the area of the excitation light passing through the sample surface and the size of the sample when the observation was performed in (**c**) shallow regions and (**d**) deep regions. The yellow circle indicates the area through which the excitation light passes and the red dotted box indicates the range of pre-scanning. (**e**) $xy$ image at a depth of 210 μm from the measurement start position. Scale bar indicates 200 μm. (**f**) Calculated surface shape of the sample.

We confirmed the effectiveness of the ACMSS using a biological sample. Figure 5(a,b) show photographs of the observed mouse brain. The blood vessels of the mouse brain were stained with DiI fluorescent dye^35,36^, and the mouse brain was cleared by an optical clearing agent called SeeDB^18^ (${n}_{2}$ = 1.47) to clarify the aberration correction effect.

To elucidate the surface shape of the mouse brain, we performed pre-scanning and measured the auto-fluorescence of the sample. The measurements were performed near the position indicated with a blue arrow in Fig. 5(a). In Fig. 5 (c), when observing shallow regions, the passage area of the excitation light on the sample surface is narrow. However, in observation at deeper regions, for example, when there is an objective lens with NA 0.7 at a position of $d$ = 1200 µm from the interface, the excitation light passes through a circle with a diameter of 2400 μm on the sample surface (Fig. 5(d)). Figure 5(d) also shows the relationship between the passage area of the light and the size of the brain. The sample surface in the area through which light passes is curved. Therefore, we performed observation in an area of 2,576 × 2,576 μm^2^ in pre-scanning to clarify the surface shape through which the excitation light passes. Figure 5(e) shows the $xy$ image at a depth of 210 μm from the measurement start position. The sample surface (the interface between air and the sample) is confirmed by auto-fluorescence. Edge detection is performed from the image obtained at each depth, and the three-dimensional position of the sample surface is obtained. Figure 5(f) shows the surface shape of the sample expressed as a polynomial approximation. As shown in Fig. 5(f), the sample has a curved surface, and the maximum height difference is approximately 1,000 μm.

**Figure 6:**
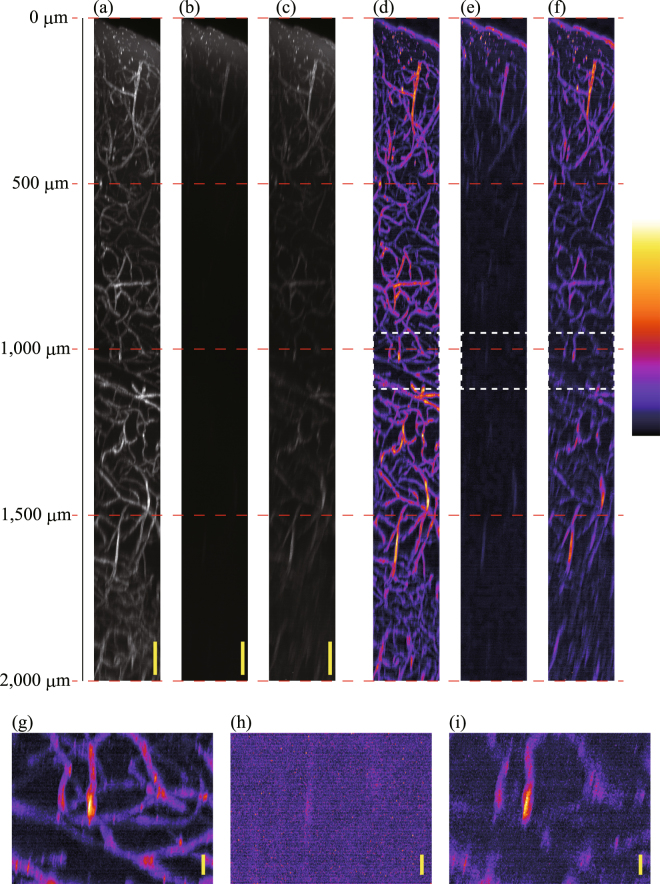
Observation results of blood vessels in a mouse cerebrum. (**a**)–(**c**) $xz$-projected images for an optical depth of 0 μm to 2,000 μm from scans performed with wavefront modulation using the ACMSS, with wavefront modulation using the SACM, and without wavefront modulation, respectively. (**d**)–(**f**) False-colour images with gamma correction and background subtraction of (**a**–**c**). (**g**–**i**) Magnified $xz$-projected images from 950 to 1,100 μm. Scale bars indicate 100 μm in (**a**–**c**), and 20 μm in (**g**–**i**).

Figure 6(a–c) show the $xz$-projected images obtained using the maximum fluorescence intensity of the blood vessels in the mouse cerebrum from an optical depth of 0 μm to 2,000 μm when a TPM scan was performed with wavefront modulation using ACMSS, with wavefront modulation using SACM, and without wavefront modulation, respectively. The excitation light intensity was changed depending on the observation depth (Supplementary Figure 4). The projected images were formed using the acquired two-dimensional images with no other image processing except for intensity normalisation. Three-dimensional images are provided as Supplementary Video 1. The brightness of each image was normalised using the maximum fluorescence intensity of the blood vessels from the scan with wavefront modulation using the ACMSS. To enhance the visibility of Fig. 6(a–c), false-colour images with gamma correction and background subtraction are shown in Fig. 6(d–f). Moreover, Fig. 6(g–i) show the magnified $xz$-projected images of Fig. 6(d–f) from 950 to 1,100 μm. In Fig. 6(i), although thick blood vessels extending in the depth direction can be observed, other blood vessels, particularly blood vessels extending in the direction perpendicular to the depth direction, cannot be observed. Thus, the state of the vascular pattern is not clear. On the other hand, in Fig. 6(g), the vasculature is clearly observed. Finally, we observed the blood vessels up to 2,000-μm optical depth using the ACMSS.

**Figure 7:**
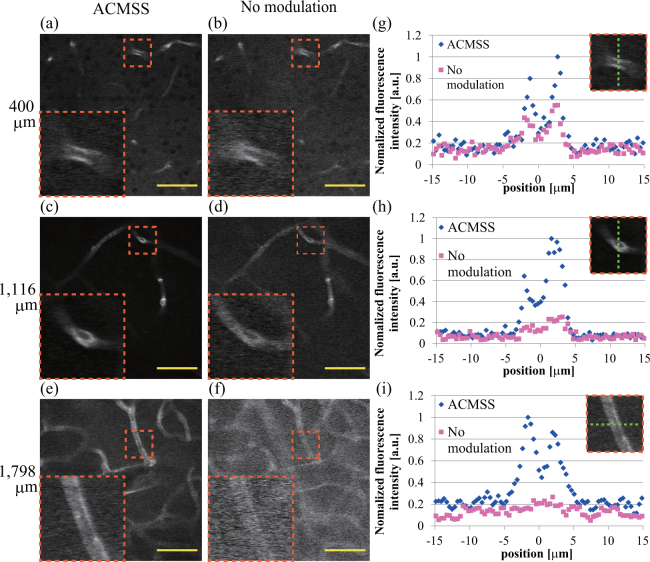
Observation of $xy$ images of blood vessels in a mouse cerebrum. (**a**,**b**) $xy$ image at an optical depth of 400 μm from the scans performed with wavefront modulation using the ACMSS and without wavefront modulation. (**c**,**d**) $xy$ images at an optical depth of 1,116 μm. (**e**,**f**) $xy$ images at an optical depth of 1,798 μm. Scale bar indicates 50 μm. Magnified views of the blood vessels in the orange dashed box are shown in the lower left of (**a**–**f**). (**g**)–(**i**) Fluorescence intensity of the blood vessels across the green dashed line in the orange dashed box.

Figures 7(a,b) show the $xy$ images at an optical depth of 400 μm with wavefront modulation using the ACMSS and without wavefront modulation, respectively. Similarly, the $xy$ images at depths of 1,116 μm and 1,798 μm are shown in Fig. 7(c–f). The brightness of each image is normalised by the respective maximum intensity. The magnified view of the blood vessel in the orange dashed box is shown on the lower left of Fig. 7(a–f). The line profiles of these blood vessels are indicated in Fig. 7(g–i), respectively. We confirmed that the signal-to-noise ratio is improved by wavefront modulation using the ACMSS. After subtracting the background, the maximum fluorescence intensity with wavefront modulation using the ACMSS is approximately 2.0, 5.5, and 15.3 times higher than that without wavefront modulation at optical depths of 400, 1,116, and 1,798 μm, respectively.

**Figure 8:**
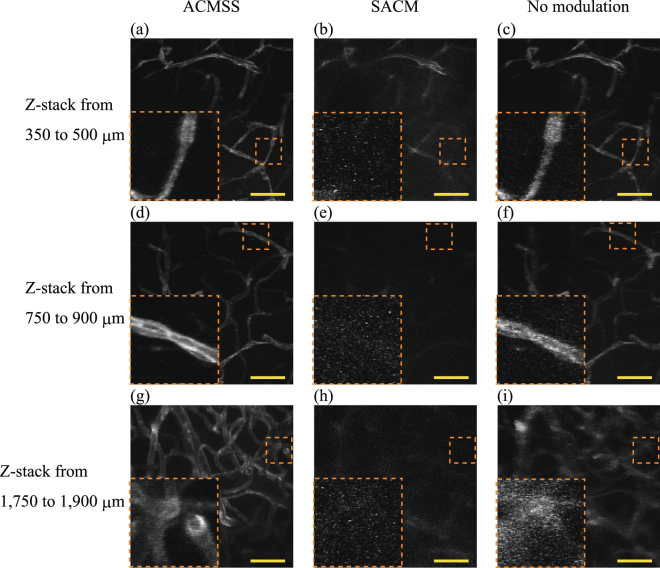
Maximum-intensity *z*-projection images. (**a**–**c**) *z*-projection images from an optical depth of 350 μm to 500 μm from the scan performed with wavefront modulation using ACMSS, with wavefront modulation using SACM, and without wavefront modulation, respectively. (**d**–**f**) $z$- projection images from 750 μm to 900 μm. (**g**–**i**), $z$- projection images from 1,750 μm to 1,900 μm. Scale bar indicates 50 μm. Magnified views of the vessels in the orange dashed box are shown in the lower left of (**a**–**i**). The brightness of each image is normalised using the maximum fluorescence intensity of each image after background-noise subtraction.

Figure 8(a–c) show the maximum-intensity $z$-projection images from an optical depth of 350 μm to 500 μm from the scan performed with wavefront modulation using the ACMSS, with wavefront modulation using the SACM, and without wavefront modulation, respectively. Similarly, the maximum-intensity $z$-projection images at depths from 750 μm to 900 μm and from 1,750 μm to 1,900 μm are shown in Fig. 8(d–i). By employing the ACMSS, individual vascular endothelial cells^36^ appear distinctly in the $z$-projection image composed of the images obtained from optical depths from 750 μm to 900 μm (Fig. 8(d)). From Fig. 8(i), it can be seen that the blood vessels were blurred when the scan was performed without wavefront modulation. On the other hand, for the scan performed with the ACMSS, the structure of blood vessels could be confirmed (Fig. 8(g)).

## Discussion

**Figure 9:**
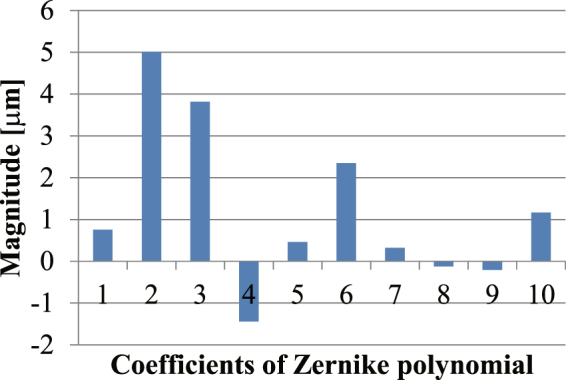
Coefficients of the Zernike polynomial of the aberration when the blood vessels at 1,000-μm optical depth in a mouse were observed. The horizontal axis represents the index of the Zernike polynomial. Indices 1 and 2 are tilt, 3 is defocus, 4 and 5 are astigmatism, 6 and 7 are coma, 8 and 9 are trefoil, and 10 is spherical aberration.

The pre-distortion wavefront using ACMSS and the sample aberration have a conjugate relationship. Thus, we expressed the aberration at an optical depth of 1,000 μm in the biological sample as a Zernike polynomial, and the result is shown in Fig. 9. The excitation light was affected by tilt, astigmatism, coma, and a spherical aberration, and these aberrations were corrected by wavefront modulation using ACMSS.

The area where aberration is well corrected is shifted by the residual coma. The shift of the area can also be confirmed from Figs 2(d) and 3(d). In these results, the deepest region of the observable bead is at the left end. On the other hand, in Fig. 5(b), the area is away from the field of view since the residual coma is too large.

In order to confirm the effect of ACMSS, we compared it with the observation using sensor-less AO^24,27^. We observed the fluorescent beads in the spherical-crown-shaped transparent epoxy resin. First, we performed sensor-less AO and found the coefficients of the Zernike polynomial that maximise the fluorescence intensity. Subsequently, scanning with wavefront modulation using the ACMSS was performed, and the maximum intensity and Zernike coefficients were obtained. The results of ACMSS almost correspond with those of sensor-less AO. In sensor-less AO, multiple scans were performed for calibration, but in the ACMSS, the scan for calibration need not be performed after the measurement of the sample surface shape. The ACMSS provided a TPM image of high quality; nonetheless, the computation time for generating the pre-distortion wavefront was approximately 1 s, which could have been improved by PC performance enhancement. In this study, a dry objective lens was used; however, an immersion-fluid objective lens is also applicable to the observation (Supplementary Figure 5).

The ACMSS corrects for predictable aberrations caused by the surface shape. The aberrations are constructed by low-order aberrations that strongly affect the fluorescence intensity. The proposed method can be expected to correct aberrations caused by the internal structure and high-order aberrations in combination with AO. Even with AO, it is difficult to directly observe the depth at which the fluorescence intensity from the guide star becomes smaller than the background noise because of the large aberration of the excitation light. When a wavefront sensor is used, the fluorescence intensity entering each segment tends to be weakened because the fluorescence is divided and converged for each segment. Previously, a method using the excitation light for two-photon excitation as a guide star has been proposed^23^. If the excitation intensity is increased excessively in order to increase the fluorescence intensity of the guide star, the sample becomes photo-damaged and bleached. By correcting the predictable aberrations using the ACMSS as a pre-processing step, the guide star is expected to be observed with an excitation light intensity that does not damage the sample. In AO without a wavefront sensor, we expect that our method would reduce the number of scans for the calibration of AO, thereby shortening the measurement time.

We believe it is effective to perform AO^21–28^ and a multiplexed aberration measurement method^37^ after obtaining the fluorescence intensity of the guide star buried in the background noise by employing the ACMSS.

The novelty of our study is that we derived a simple OPD calculation method for correcting aberration caused by the curved sample surface shape, and we realised deep observation at the cellular level even with a dry objective lens. Moreover, the proposed method reduces the restrictions on the placement and observation position when observing a sample with a curved surface. For observation at a greater depth, excitation light having a higher power and longer wavelength^15,38^ may be adopted.

## Methods

### Optical Setup

The optical setup was equivalent to the one in ref.^39^ (Supplementary Figure 6). A Ti:sapphire laser (Chameleon Vision II, Coherent, Inc.) was used to deliver horizontally polarised light to a beam expander. A femtosecond train of optical pulses (880-nm wavelength, 150-fs pulse duration, 80-MHz repetition rate) was projected onto a liquid crystal on a silicon-type spatial light modulator (LCOS-SLM, 1280 × 1024 pixels, 12.5-μm pixel pitch, 700-nm to 1000-nm bandwidth multi-layered dielectric mirror, Hamamatsu Photonics K.K.) with a Peltier system^40^. The wavefront of the excitation light was modulated to a pre-distortion wavefront for aberration correction according to a hologram applied to the SLM. The light was reflected and directed through one telecentric lens system to an $xy$ galvo scanning system (6220 H, Cambridge Technology), which was coupled to a second telecentric lens system. The light, with its incident angle varied using the scanner, was then directed to a dry objective lens (UCPLFLN 20x magnification, NA 0.7, 1800-μm working distance, Olympus) by a third telecentric lens system. These telecentric lens systems were used to ensure that the wavefront of the light was transmitted from the plane of the SLM to the pupil plane of the objective lens in an upright microscope system. The light was focused onto the sample using an objective lens in order to excite fluorescence, which was then collected by the objective lens. The collected fluorescence was de-scanned by the $xy$ galvo system, and the lens was focused onto the photo-multiplier tube (PMT; 10771P-40, Hamamatsu Photonics K.K.). The two-dimensional image ($xy$ image) was formed from fluorescence detected by the PMT. To obtain the three-dimensional image, scans were performed at each depth by moving the objective lens. In the experiment using fluorescent beads of 0.20-μm size, the lateral and axial full width at half maximum of the point spread function of the system were 1.01 μm and 2.96 μm, respectively.

### Evaluation of the Observation Results

To quantify the improvement in the fluorescence intensity from the fluorescent beads, an improvement ratio was calculated as the ratio between the fluorescence intensity from the scan with wavefront modulation and without wavefront modulation, which can be expressed as follows:

7 $${{\rm{Ratio}}}_{{\rm{experiment}}}(x{,}y{,}z)=\frac{{{Q}}_{\mathrm{with}\_\mathrm{wavefront}\_\mathrm{modulation}}(x{,}y{,}z)}{{{Q}}_{\mathrm{without}\_\mathrm{wavefront}\_\mathrm{modulation}}(x{,}y{,}z)},$$

where ${Q}_{\mathrm{with}\_\mathrm{wavefront}\_\mathrm{modulation}}$, ${Q}_{\mathrm{without}\_\mathrm{wavefront}\_\mathrm{modulation}}$ are the averages of the fluorescence intensities in a region of interest (ROI) (central coordinates are (*x, y, z*)) in a bead when a scan with and without wavefront modulation was performed at an observation depth of $z$μm. Here, the ROI was chosen as a square area of side 3 pixels on the two-dimensional image around the centre of gravity of each bead.

### Animal Procedures

We purchased 8-week-old male C57BL/6 J mice from Japan SLC (Shizuoka, Japan). The blood vessels in the mouse brain were stained with DiI, as previously described^32^, following which the brain was cleared with SeeDB^18^. All animal experiments were performed with the approval of the Institutional Animal Care and Use Committee of Hamamatsu University School of Medicine (Shizuoka, Japan) in accordance with their guidelines.

### Surface Shape Measurement Using Auto-Fluorescence

The sample surface shape can be clarified by obtaining the three-dimensional position of the sample surface and expressing it by polynomial approximation. Near the sample surface, the excitation light is only slightly influenced by aberrations; therefore, pre-scanning without wavefront modulation was performed. We performed the pre-scanning within an area of 2,576 × 2,576 μm^2^ in the direction perpendicular to the optical axis (the $x$-direction and $y$-direction). After the measurement was performed at a certain depth, the dry objective lens was moved in the optical-axis direction ($z$-direction), and a two-dimensional image at a different depth was obtained. The three-dimensional image was formed from multiple two-dimensional images.

### TPM Scan

We carried out scans from the surface of the sample to an optical depth of 2,000 μm with the objective lens positioned at 1-μm increments. The length in the $xy$ direction was 200 μm. At each depth, we performed scans in the following order: without wavefront modulation, with wavefront modulation using SACM, and with wavefront modulation using ACMSS. In the case of the scans with wavefront modulation, the pre-distortion wavefront was calculated, and the wavefront of the excitation light was modulated every 1 μm that the objective lens was moved. After scanning, we constructed a three-dimensional image from the two-dimensional $xy$ images. The observed depth was indicated by the optical depth. When the scan was performed without wavefront modulation using a dry objective lens, the optical depth was derived by multiplying the distance $d$ from the interface with the refractive index ${n}_{2}$.

## Electronic supplementary material

Supplementary Information

Supplementary Video 1
