# Supplementary material for: Aberration correction considering curved sample surface shape for non-contact two-photon excitation microscopy with spatial light modulator
Source: Sci Rep. 2018 Jun 18;8:9252. doi: 10.1038/s41598-018-27693-7 (PMC6018692; doi:10.1038/s41598-018-27693-7)
Supplement: Supplementary file 1 — Supplementary Information [file 41598_2018_27693_MOESM1_ESM.docx]

**Supplementary Information**

**Aberration correction considering curved sample surface shape for non-contact two-photon excitation microscopy with spatial light modulator**

Naoya Matsumoto^1^, * ^, +^, Alu Konno^2, +^, Takashi Inoue^1^, and Shigetoshi Okazaki^2^

^1^Central Research Laboratory, Hamamatsu Photonics K.K., Shizuoka, Japan

^2^Department of Medical Spectroscopy, Preeminent Medical Photonics Education & Research Center, Institute for Medical Photonics Research, Hamamatsu University School of Medicine, Shizuoka, Japan

*Correspondence: nm@crl.hpk.co.jp

+These authors contributed equally to this work.


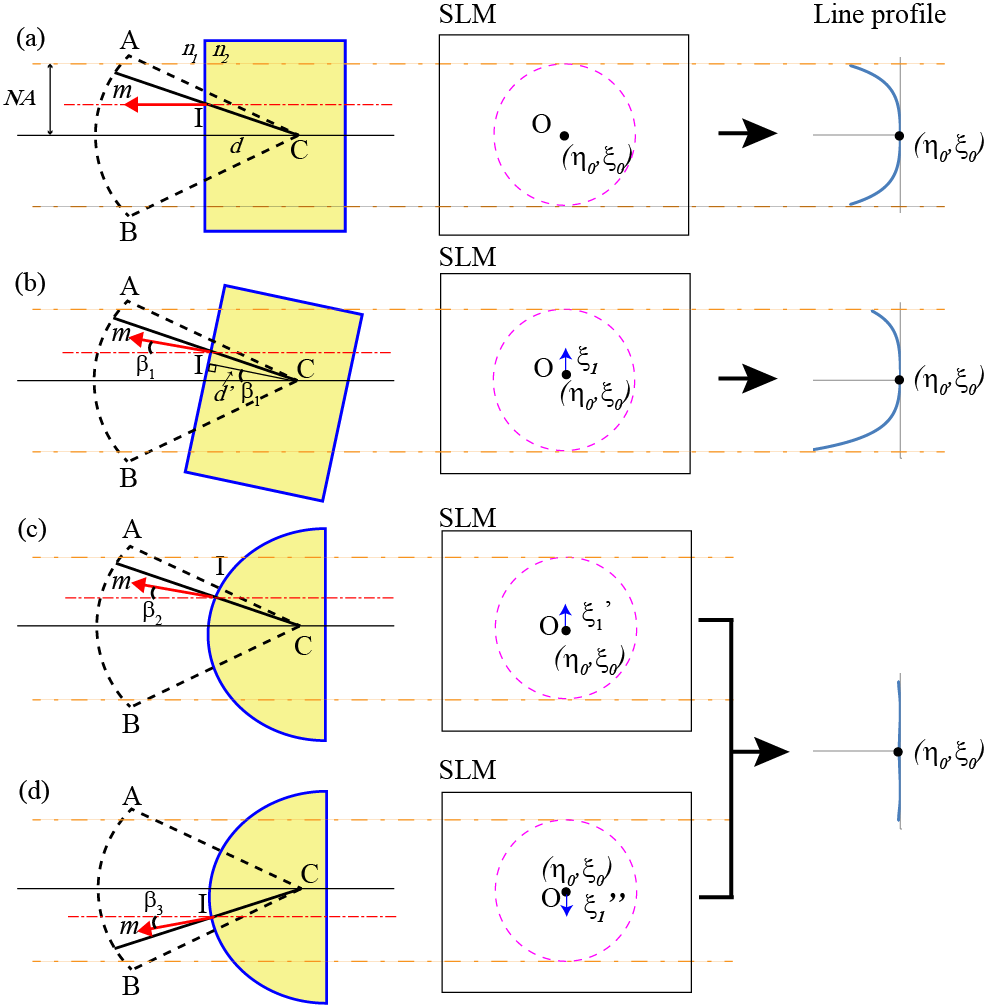


**Supplementary Figure 1.** Conceptual diagram of the proposed method. (a) Conventional spherical aberration correction method: the interface between air and the sample is a plane perpendicular to the optical axis. The arch AB represents the reference spherical wavefront when a collimated laser beam is incident on the objective lens. The direction of the normal vector (red arrow) at the intersection point of the sample and the ray forming the excitation light corresponds to the optical axis. At this time, the centre of the designed pre-distortion wavefront corresponds to the centre of the objective lens and the centre ($\eta_{0}$,$\xi_{0}$) of the light irradiated on the SLM. (b) Proposed aberration correction method for the sample tilt: the interface between air and the sample is tilted by $\beta_{1}$ rad with respect to the optical axis. The movement of the pre-distortion wavefront is derived according to the tilt of the normal vector with respect to the optical axis. At this time, the centre of the pre-distortion wavefront moves from the centre ($\eta_{0}$,$\xi_{0}$) of the light irradiated on the SLM to the position ($\eta_{0}$,$\xi_{0}+\xi_{1}$). The movement $(\eta_{1},\xi_{1})$ of the pre-distortion wavefront was obtained by applying $\beta_{1}$ in equations (3) to (6). (c)–(d) Proposed aberration correction method considering the curved sample surface shape (ACMSS): when the sample has a curved surface, it has a normal vector with a different tilt at each intersection of the ray forming the excitation light and the sample surface. The pre-distortion wavefront for aberration correction can be obtained by applying a different tilt in equations (3) to (6) at each intersection point. The line profiles of the pre-distortion wavefront are also shown.


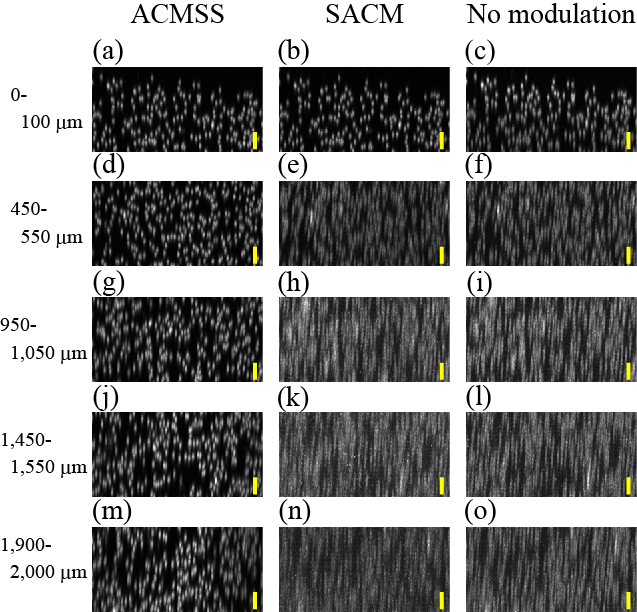


**Supplementary Figure 2.** Observation results of 3-μm-diameter fluorescent beads in a transparent epoxy resin tilted at 0.0873 radian (5°). (a)-(c) Magnified $xz$-projected images of Figures 2(c)–(e), respectively, from 0 μm to 100 μm. Similarly, magnified $xz$-projected images from 450 to 550 μm, from 950 μm to 1,050 μm, from 1,450 μm to 1,550 μm, and from 1,900 μm to 2,000 μm are shown in (d)-(f), (g)-(i), (j)-(l), and (m)-(o), respectively. Scale bars indicate 20 μm.


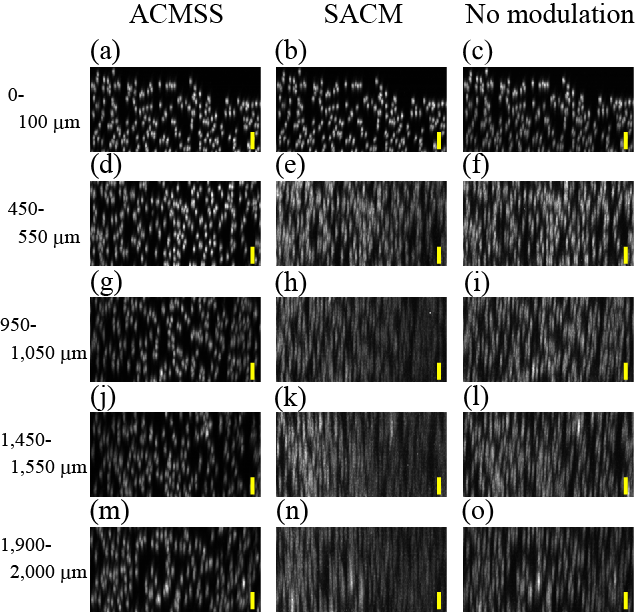


**Supplementary Figure 3.** Observation results of 3-μm-diameter fluorescent beads in a spherical-crown-shaped transparent epoxy resin (radius of 6 mm). (a)–(c) Magnified $xz$-projected images of Figures 3(c) to (e), respectively, from 0 μm to 100 μm. Similarly, the magnified $xz$-projected images from 450 μm to 550 μm, from 950 μm to 1,050 μm, from 1,450 μm to 1,550 μm, and from 1,900 μm to 2,000 μm are shown in (d)-(f), (g)-(i), (j)-(l), and (m)-(o), respectively. Scale bars indicate 20 μm.


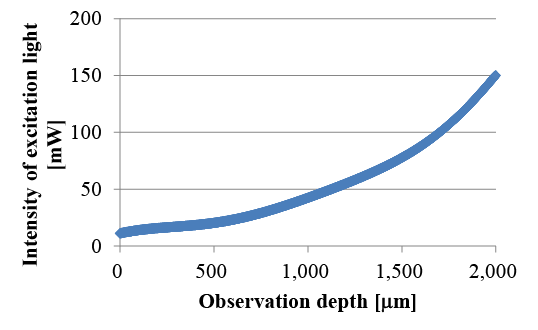


**Supplementary Figure 4.** Relationship between excitation light intensity and observation depth in the biological sample. To clarify the effect of the aberration correction in deeper regions, the intensity of the excitation light is changed depending on the observation depth. The intensity of the excitation light was measured under the objective lens.


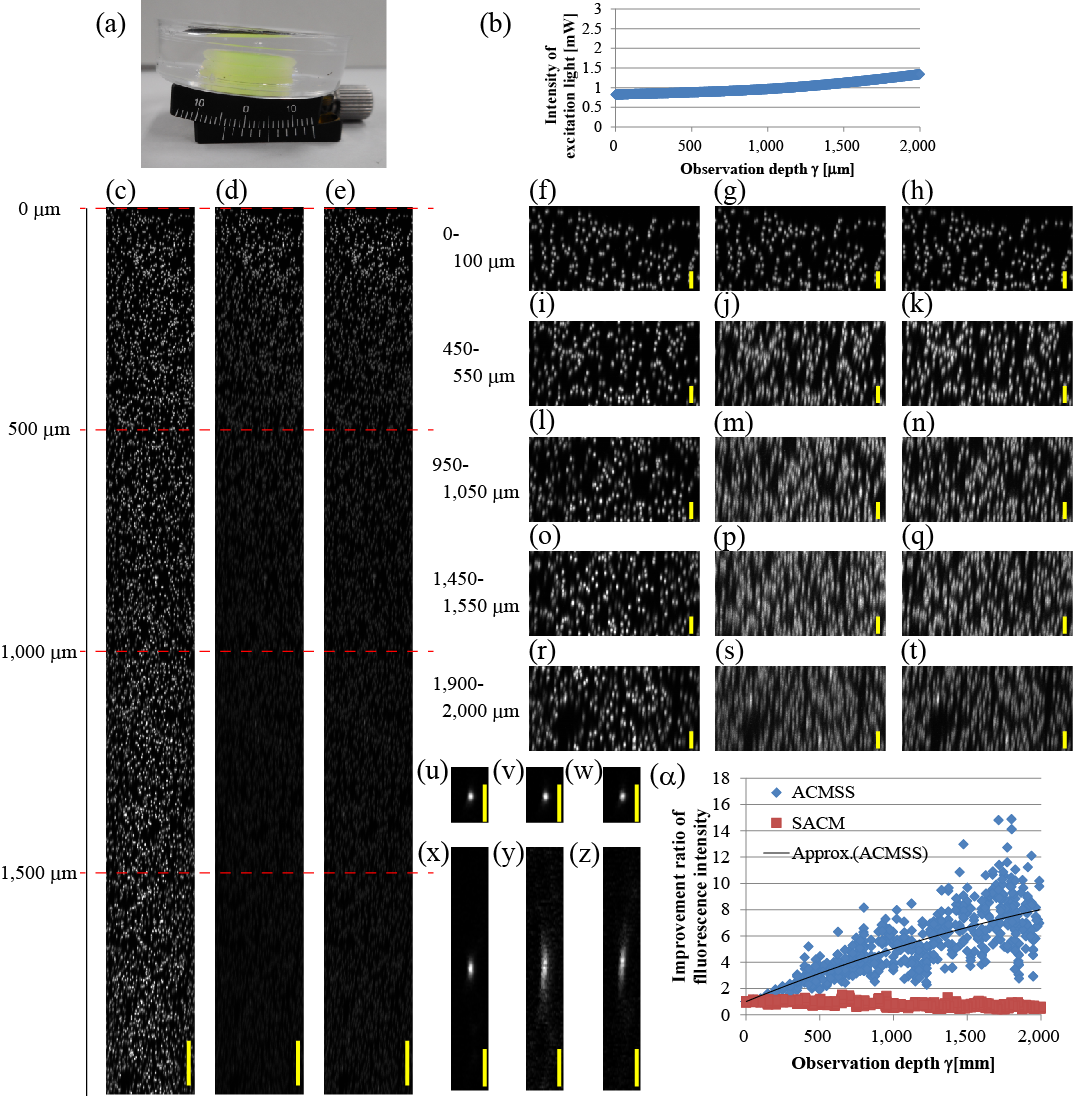


**Supplementary Figure 5.** Observation results of fluorescent beads of 3-μm diameter in a transparent epoxy resin, which was tilted at 0.0873 rad (5°) by using a water immersion lens (XLUMPLFLN 20× magnification, NA 1.0, 2,000-μm working distance, Olympus). (a) Photograph of the transparent epoxy resin tilted by a goniometric rotation stage. (b) Excitation light intensity. To clarify the effect of the aberration correction in the deeper regions, the excitation light intensity was changed depending on the observation depth. The excitation light intensity was measured under the objective lens. (c)-(e) $xz$-projected images for an optical depth of 0 μm to 2,000 μm from the scans performed with wavefront modulation for the sample tilt, with wavefront modulation using SACM, and without wavefront modulation, respectively. (f)–(h) Magnified $xz$-projected images of Supplementary Figures 5(c)–(e), respectively, from 0 μm to 100 μm. (i)-(t) Similarly, magnified $xz$-projected images from 450 μm to 550 μm, from 950 μm to 1,050 μm, from 1,450 μm to 1,550 μm, and from 1,900 μm to 2,000 μm. (u)-(w) $xz$ image of the observed bead at 37-μm optical depth when TPM scan was performed with wavefront modulation for the sample tilt, with wavefront modulation using SACM, and without wavefront modulation, respectively. (x)-(z) $xz$ image of the observed bead at 1,946-μm optical depth. (*α*) Quantitative evaluation of the improvement in the fluorescence intensity from the fluorescent beads. Scale bars indicate 100 μm in Supplementary Figures 5(c)-(e) and 20 μm in Supplementary Figures 5(f)-(z).


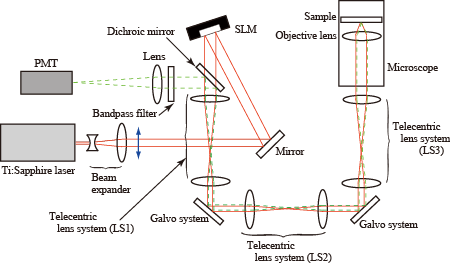


**Supplementary Figure 6.** Schematic of the experimental two-photon excitation microscopy system using an SLM. The solid line (red) and dashed lines (green) represent the excitation beam and fluorescence, respectively. By changing the CGH applied to the SLM, the system can perform three types of scans: scan with wavefront modulation using ACMSS, scan with wavefront modulation using SACM, and scan without wavefront modulation. The blue double-head arrow indicates the polarization direction of the excitation beam.


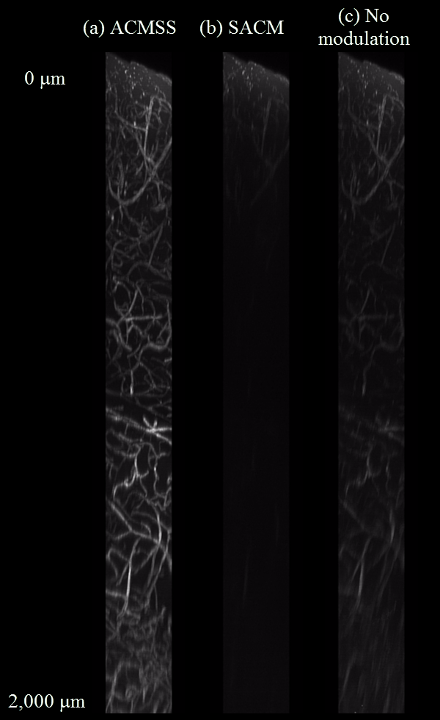


**Supplementary Video 1.** Three-dimensional image of blood vessels in a mouse cerebrum for an optical depth of 0 μm to 2,000 μm from scans performed with wavefront modulation using ACMSS, with wavefront modulation using SACM, and without wavefront modulation.
